# A Meta-Analysis of Adalimumab for Fistula in Crohn's Disease

**DOI:** 10.1155/2017/1745692

**Published:** 2017-10-24

**Authors:** Yin-mei Fu, Ming Chen, Ai-jun Liao

**Affiliations:** Department of Gastroenterology and Hepatology, First Affiliated Hospital of University of South China, Hengyang, Hunan 421001, China

## Abstract

**Purpose:**

This study aimed to evaluate the therapeutic value of adalimumab (ADA) for fistula in Crohn's disease (CD).

**Methods:**

A computerized search of electronic databases, including PubMed, Web of Science, Embase, Google scholar, and the Cochrane Library from 2000 to October 2016, was performed. Randomized controlled trials (rcts) or nonrandomized controlled trials (n-rcts) were included in this article to evaluate the role of ADA in the management of fistula in CD. The methodological index for nonrandomized studies (MINORS evaluation tools) was used to assess the quality of every study.

**Result:**

Overall, seven studies and 379 patients comforted to the inclusion criteria of this meta-analysis. The result showed that 36% (95% CI: 0.31–0.41) of patients with complete fistula closure and 31% (95% CI: 0.031–0.61) of patients with partial response were received in CD with ADA treatment.

**Conclusion:**

We concluded that ADA is effective and safe for the treatment of fistula in CD according to current evidence.

## 1. Introduction

Crohn's disease (CD) is a chronic inflammatory condition of the gastrointestinal tract resulting in inflammation, stricturing, and fistula secondary to transmural inflammation [1]. The considerable morbidity of CD is associated with fistulas, and up to 50% of CD patients are affected by fistulas [2]. Despite intensive medical and surgical treatments being used in CD therapy, however, perianal CD problems have a negative impact on the perceived health-related quality of life [3].

Different drugs have been used to treat the perianal fistulizing disease. One randomized trial reported that antibiotics (metronidazole and ciprofloxacin) have importantly improved the symptoms but rarely induce fistula healing completely [4]. Immunosuppressants have a role, but slow initial response, side effects, and relatively low remission rates of up to around a third with frequent recurrence limit their value [5]. Antitumor necrosis factor (anti-TNF) agents have been proven to improve the symptoms as well as heal the fistula tracts, for example, infliximab (IFX), adalimumab (ADA), certolizumab pegol (CDP870), and so on [6]. A randomized clinical trial showed that infliximab (IFX) can effectively induce and maintain the closure of perianal fistulas in CD patients [7]. In the attempt to reduce the immunogenic responses induced by chimeric antibodies, new approaches tried to remove all mouse-derived sequences, hence to develop fully human monoclonal antibodies [8–10]. ADA is a full human IgG1 monoclonal antibody to TNF that is effective in inducing and maintaining clinical response or remission in active inflammatory CD patients [11], as well as in managing the fistulas. However, these data are only available from subgroups in larger CD studies not specifically designed to assess the fistula response [12–17]. In this study, we evaluate the efficacy of ADA for fistula CD through one meta-analysis of randomized placebo-controlled trials collecting up-to-date reviews.

## 2. Methods

### 2.1. Search Strategy and Study Selection

Clinical trials were researched through PubMed, Web of Science, Embase, Google scholar, and the Cochrane Library and used the following keywords: tumor necrosis factor, anti-TNF, TNF, adalimumab, Crohn's disease, fistula, randomized, randomly, and clinical trials between 2000 and October 2016; finally, seven studies were selected, and all these studies in this meta-analysis were written in English. When multiple reports describing the same population were published, the most recent or complete report was used.

### 2.2. Inclusion and Exclusion Criteria

The inclusion criteria are the following: (1) study types—rcts, n-rcts, case-control study, and cohort study. (2) Study object: definite diagnostic criteria (medical history, clinical manifestation, colonoscopy examination, and histological examination of apricot mucosa) in patients with CD who have fistula. (3) Intervention measures—a test group to give any ANA treatment of the dosage, whether to set the control group is not limited, if the control group is set, the intervention measures are antibiotics such as metronidazole and ciprofloxacin; immunosuppressive agents such as azathioprine (AZA), 6-mercaptopurine (6MP), and methotrexate (MTX); and immunomodulators such as antagonists of tumor necrosis factor (TNF)-alpha (infliximab (IFX)), but not surgery or stem cells. (4) Outcome—assessment of efficacy of fistula closure and safety treated with ADA.

The exclusion criteria are the following: (1) studies not accessible to full research data, review, case report, letter, and editorial; articles about children or pregnant woman were also excluded, (2) CD patients without fistula or not receiving ADA treatment, and (3) the same center repeated in the same study.

Data from the included studies were extracted and summarized independently by two of the authors (Ming Chen and Yin-mei Fu). Two authors were both blinded to the writers, the institutions, and the journals of each article. Each disagreement had been solved by the senior author (Ai-jun Liao).

### 2.3. Study Quality Evaluation

Seven studies were included in the meta-analysis, most of which were nonrandomized. Few validated instruments are available to determine the methodological quality of observational or non-randomized studies, either from the reader's perspective or for the purpose of the meta-analysis. The methodological index for nonrandomized studies (MINORS evaluation tools) [18] was used to assess the quality of studies. This validated index involves 12 items, in which the first eight items were specifically designed for noncomparative studies and the remaining four items were applied to comparative studies. Items are scored as 0 (not reported), 1 (reported but inadequate), and 2 (reported and adequate). The maximum ideal score for noncomparative studies is 16, and for comparative studies, it is 24.

### 2.4. Statistical Analysis

All analyses were performed using Stata software (Stata Inc., version 12.0, USA). We used a *Q* test to assess the heterogeneity of the results of the study and *I*^2^ for the quality analysis of the heterogeneity. *Q* and *I*^2^ statistics were used to perform the heterogeneity across trials. *I*^2^ values ranged from 0 to 100% (0% suggested no observed heterogeneity, 25–49% suggested low, 50–74% moderate, and ≥75% high heterogeneity). *P* value less than 0.1 was considered significant heterogeneity. If heterogeneity existed, a random effect model was used to assess the overall estimate; otherwise, a fixed effect model was chosen.

### 2.5. Sensitivity Analysis and Publication Bias

Sensitivity analyses, which were assessed by using high-quality articles, were performed to explore the potential sources of heterogeneity and to eliminate the effect of low-quality articles. Funnel plots were used to screen for potential publication bias.

## 3. Result

### 3.1. The Retrieval Process and Study Characteristic

**Figure 1:**
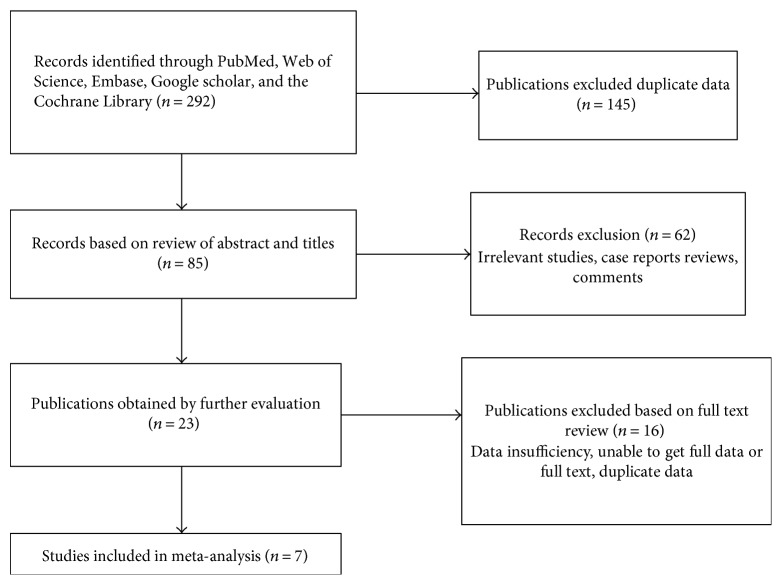
Document retrieval process.

There were 292 relevant articles through PubMed, Embase, Web of Science, the Cochrane Library, and Google scholar. EndNote software was used to eliminate duplicate documents, and the authors considered ten studies after full-text review and eventually included seven studies in the meta-analysis [12, 13, 15–17, 19, 20] (Figure 1()). Two trials were excluded as they have less sample size [14, 21]. During the whole retrieval process, five relevant studies could not be accessed in full text and full data [22–26]. A total of 379 patients with CD and fistula were proved to have ADA treatment. The studies included two trial designs: complete closure fistula rate and partial response rate. All the studies were published in English. The characteristics and the methodological quality of the studies included in the meta-analysis are summarized in Table 1.

### 3.2. Efficacy of Adalimumab on Complete Fistula Closure in Crohn's Disease

It is important to assess complete fistula closure by adalimumab treatment in Crohn's disease (Table 2). The trials by Hinojosa et al. [16], Colombel et al. [13], Lichtiger et al. [17], Dewint et al. [15], Castaño-Milla et al. [12], Panaccione et al. [27], and Rizzello et al. [20] were included in the meta-analysis.

The first study by Hinojosa et al. [16] was a prospective multicenter, open-label, observational, 52-week study. All patients received an induction dose of adalimumab (160 mg at baseline followed by 80 mg at week 4). However, it was reported that the number of fistulas were draining at baseline at week 4. So there was 23% complete closure of all fistulas at week 4.

This study by Colombel et al. [13] was a phase III, multicenter, randomized, double-blind, placebo-controlled study with an open-label extension in 92 sites. All patients received initial open-label adalimumab induction therapy (80 mg/40 mg at weeks 0/2). At week 4, all patients were randomly assigned to receive double-blind placebo or adalimumab 40 mg every other week or weekly to week 56 (irrespective of fistula status). It was published that there was 40% complete fistula closure at week 56.

The CHOICE trial was written by Lichtiger et al. [17] to evaluate safety, fistula healing, the quality of life, and work productivity in adalimumab-treated patients who failed infliximab treatment, including primary nonresponders. After 8-week infliximab washout, patients with moderate-to-severe Crohn's disease received open-label adalimumab as induction (160/80 mg at weeks 0/2) and maintenance (40 mg every other week) therapies. At/after 8 weeks, patients with flare/nonresponse could receive weekly therapy. Approximately 40% of patients (34 of 88 patients) had complete fistula healing at the last visit.

The other trial by Dewint et al. [15] was a randomized, double-blind, placebo-controlled trial in eight Dutch hospitals. After adalimumab induction therapy (160/80 mg at weeks 0/2), patients received 40 mg every other week twice daily for 24 weeks. 100% reduction was 33% at 24 weeks.

The fifth study by Castaño-Milla et al. [12] was a retrospective multicenter study to assess the effectiveness of ADA in the treatment of perianal fistulas in CD patients naive to anti-TNF therapy. Eligible patients received ADA (Humira; Abbott Laboratories) 160 mg at week 0 and 80 mg at week 2, or ADA 80 mg at week 0 and 40 mg at week 2 as induction doses followed by 40 mg every other week at 12 months. 41% remission was reported at 12 months.

The sixth study by Panaccione et al. [27] was a phase III, multicenter, open-label study of patients with moderate-to-severe CD conducted at 42 sites in Canada from January 27, 2007, to January 10, 2008. Patients received open-label adalimumab as induction (160 mg and 80 mg subcutaneously (sc)) and maintenance (40 mg sc every other week) therapy at weeks 0 and 2, respectively. If flare or nonresponse (as determined by the investigator) occurred while the patient was receiving 40 mg sc eow, the regimen could be changed to 40 mg sc weekly at or after week 8. 40% (27 of 68) had no draining fistulas at week 24.

The seventh study by Rizzello et al. [20] was to evaluate the effectiveness of adalimumab in the treatment of active and perianal CD. All patients received 160/80 mg as an induction dose followed by 40 mg eow if they are responders. In case of loss of response, a weekly treatment was performed. Immunosuppressive therapy was suspended at the start of the treatment, steroid dose was reduced of 2.5 mg/week after the induction phase at week 24, and 11/46 patients (24%) were in remission.

**Figure 2:**
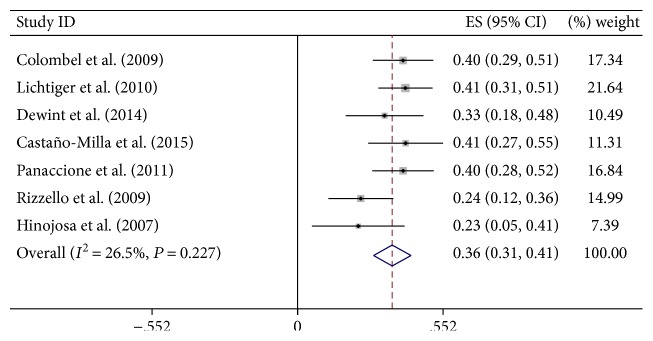
The forest plot of complete closure rate of ADA treatment on CD with fistula.

There was low significant heterogeneity in complete fistula closure rate among the studies when the data were pooled for the analysis; therefore, a fixed effect model of analysis, which assumed that the true effect size was the same in all studies, was used. The overall analysis revealed that adalimumab contributed to complete fistula closure in CD (ES 36%, 95% CI: 0.31–0.41, *I*^2^ = 26.5%, *P* = 0.000.1) (Figure 2).

### 3.3. Partial Response of Adalimumab on Draining Fistula in Crohn's Disease

In clinical practice, not every patient can achieve complete closure fistula, so goals for partial response are also important. Three studies [12, 15, 16] were reported to assess the partial response rate of adalimumab on fistula closure in Crohn's disease (Table 3); 50% reduction or partial respone was defined as ≥50% decrease in the number of fistulas that were draining at baseline.

There was a multicenter, prospective, open-label, observational, 52-week study by Hinojosa et al. [16] Patients received an induction dose of adalimumab (160 mg at baseline followed by 80 mg at week 2). However, only analyses of the short-term (4 weeks) results were reported.

The other trial by Dewint et al. [15] was a randomized, double-blind, placebo-controlled trial in eight Dutch hospitals. After adalimumab induction therapy (160/80 mg at weeks 0/2), patients received 40 mg every other week twice daily for 24 weeks. Follow-up was 24 weeks. The least 50% reduction of the number of draining fistula by ADA treatment is 47%.

The third study by Castaño-Milla et al. [12] was a retrospective multicenter study to assess the effectiveness of ADA in the treatment of perianal fistulas in CD patients naive to anti-TNF therapy. Eligible patients received ADA (Humira; Abbott Laboratories) 160 mg at week 0 and 80 mg at week 2 or ADA 80 mg at week 0 and 40 mg at week 2 as induction doses followed by 40 mg every other week. At 12 months, only 8% partial response was reported. So there were only three studies included in the meta-analysis of the partial response rate of ADA treatment on CD and fistula.

**Figure 3:**
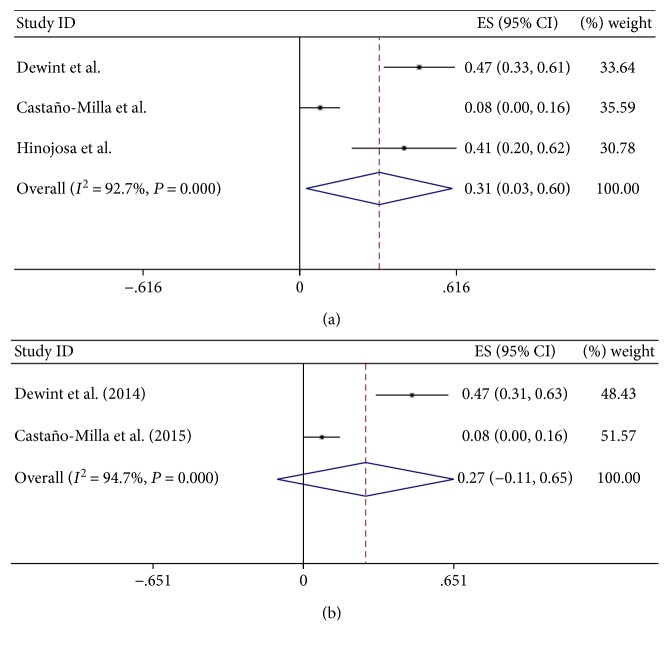
(a) The forest plot of Partial respone rate of ADA treatment on CD with fistula. (b) Sensitivity analysis of high heterogeneity about partial respone rate of ADA treatment on CD with fistula.

There was clearly high significant heterogeneity in the partial response rate of fistula in CD with ADA treatment using a fixed-effect model; authors observed statistical significance (Figure 3(a)) (ES 31%; 95% CI: 0.031–0.61; *I*^2^ = 92.7%; *P* = 0.000, <0.1). For high significant heterogeneity, the authors made sensitivity analysis that the trial by Hinojosa et al. [16] was excluded for too short therapy time. However, it was still high significant heterogeneity (Figure 3(b)) (*I*^2^ = 94.7%; *P* = 0.001, <0.1). So authors guessed that fewer trials brought about heterogeneity.

### 3.4. Safety of Adalimumab Treatment in Crohn's Disease with Fistula

**Figure 4:**
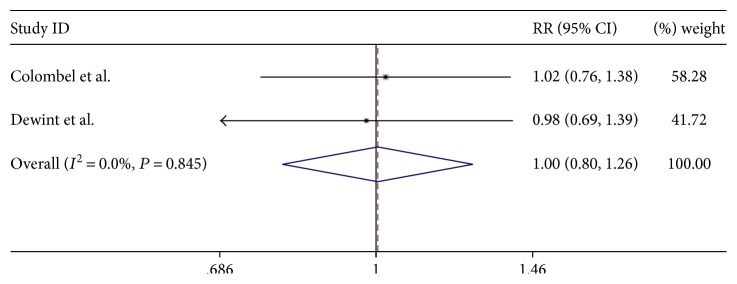
The forest plot of AEs about ADA treatment on CD and fistula.

We analyzed the incidence of adverse events (AEs). Five trials had reported the safety of ADA, and the AEs included infections, injection site reactions, abdominal tenderness, nausea, flatulence, nasopharyngitis, pharyngitis, and headache. The appearance of AEs during these two trials [13, 15] is in Table 4. It showed the lack of statistical difference between ADA therapy in CD and fistula compared with the placebo (Figure 4; *P* = 0.975, >0.1). At the same time, ADA did not also increase the risk of common adverse events by this article [11].

### 3.5. Publication Bias

**Figure 5:**
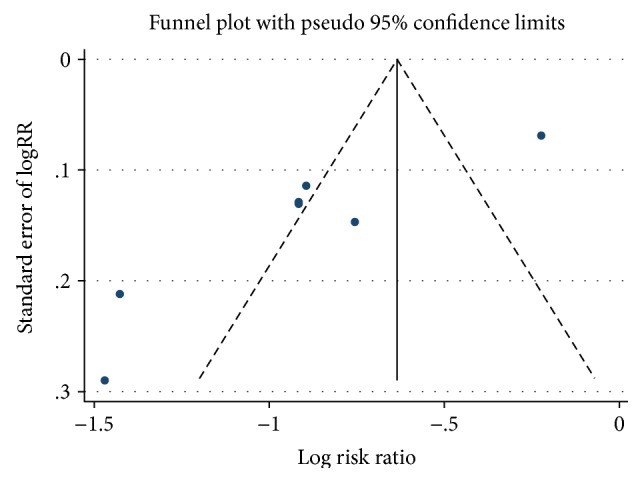
Funnel plot of adalimumab on complete fistula closure rate in CD.

**Figure 6:**
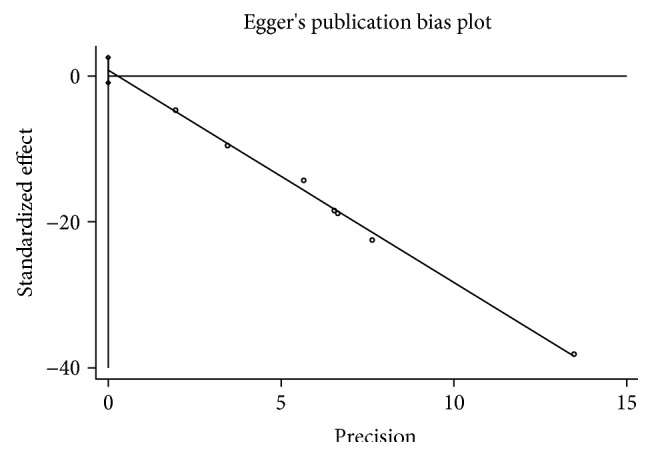
Egger plot of adalimumab on complete fistula closure rate in CD.

The seven studies were drawn by software Stata12 as a funnel plot which showed dissymmetry (Figure 5). The Egger plot (Figure 6) was used to test if the dissymmetry of the funnel plot was significant or not by metabias command; it showed that Egger regression line was through 0 point indicating that study distribution was symmetric and there is no publication bias.

## 4. Discussion

CD is a group of idiopathic chronic inflammatory intestinal disease that can damage any part of the gastrointestinal tract, mostly the ileum and colon. The prevalence of CD appears to be higher with time [28]. Fistulas, a hallmark of CD, are one of the most severe intestinal complications of CD which decreased the quality of life and increased the likelihood of total colectomy, even canceration. Medical treatment is the first choice for people with CD and fistula. Anti-TNF agents are effective as both induction and maintenance therapies in moderate-to-severe Crohn's disease in adults, including patients with fistulas. The safety profile is acceptable [29]. Compared with IFX, ADA is similar to IFX in the mode of action, efficacy, and safety, but it has advantages of causing less anaphylactic or immunological reactions [30]. Also, the previously high activity index returns to normal and the fistulas are closed. The quality of life using validated questionnaire improves significantly also [31].

According to our knowledge, this is a meta-analysis of the available published rcts or n-rcts to examine therapeutic value adalimumb on fistula closure in Crohn's disease. For the complete closure rate of ADA treatment on CD and fistula, we found that the heterogeneity across studies was not been found. In an overall analysis, the efficacy of complete fistula closure in CD by ADA treatment was 36%. It was not clear to examine the efficacy of the partial response of adalimumab on fistula healing in Crohn's disease. The few patients included may be the key to high heterogeneity about meta-analysis of efficacy of partial response of fistula healing in CD with ADA. The reasons, from a professional point of view, maybe were treatment time and fewer patients included which could not be proved. So more studies should be required to confirm those conclusions.

In addition to side effects, the rates of adverse reactions were similar in the two groups. However, these data should be noticed because of several limitations. Patients in trials might not represent patients seen in clinical practice, and follow-up might not be sufficiently long for some serious events such as malignancy to occur [11].

In the analysis, statistical heterogeneity was found in AEs; we considered that this heterogeneity may emerge for three reasons which are the number of the patients enrolled, the induction/maintenance doses for ADA among the trials, and treatment time.

There are also several limitations; we only searched the English language literature in this meta-analysis. Therefore, it is possible that they may have missed potentially relevant trials in the non-English language literature. We did not assess the factors that resulted in heterogeneity, and all studies were not rcts and a number of studies are few. However, all studies included were of high quality.

## 5. Conclusion

In summary, our findings update the previously published studies and evaluate the clinical efficacy and safety of ADA in CD patients who develop loss of response to IFX or are naive to biological treatment with perianal disease. Treatment benefit should be weighed against side effects. Although ADA for CD with fistula was largely safe, it was necessary to notice AEs. Due to the limitations of this meta-analysis, more prospective randomized trials are needed to confirm the results, especially for partial response of ADA treatment on CD with fistula. Long-term therapeutic efficacy and adverse reactions by ADA should also be concerned.

## Figures and Tables

**Table 1:** The basic characteristics of studies included in meta-analysis.

First author	Publication date	Type	*N* ^$^	Average age	Outcome	Follow-up time	Minor score
Hinojosa et al. [16]	2007	n-rct	22	37.4	23% (complete closure of all fistulas) 41% (experienced fistula improvement, 50%)	4 w	11
Colombel et al. [13]	2009	rct	70	37.1	40% (complete fistula closure)	56 w	18
Lichtiger et al. [17]	2010	n-rct	88	40.8	40.9% (complete fistula healing)	36 w	12
Dewint et al. [15]	2014	rct	39	39.3	47% : 50% reduction, 33% : 100% reduction^∗^	24 w	18
Castaño-Milla et al. [12]	2015	n-rct	46	36.5	41% remission, 8% partial response^∗^	12 m^#^	12
Panaccione et al. [23]	2011	n-rct	68	37	40% complete fistula closure	24 w^#^	12
Rizzello et al. [26]	2009	n-rct	46	35.7	24% complete fistula closure	24 w	12

^$^Case load; ^∗^100% reduction/remission: complete closure of all fistulas that were draining at baseline and 50% reduction/partial response: ≥50% decrease in the number of fistulas that were draining at baseline; ^#^m: month and w: week.

**Table 2:** Complete closure rate of ADA treatment on CD and fistula.

Trials	Therapy weeks	Complete closure fistula rate
Colombel et al.	56 w	40%
Lichtiger et al.	24 w	40.9%
Dewint et al.	24 w	33%
Castano-Milla et al.	12 m	41%
Hinojosa et al.	4 w	23%
Panaccione et al.	24 w	40%
Rizzello et al.	24 w	24%

**Table 3:** Partial response rate of ADA treatment on CD and fistula.

Trials	Therapy weeks	Partial response rate
Hinojosa et al. [16]	4 w	41%
Dewint et al. [15]	24 w	47%
Castaño-Milla et al. [12]	12 m	8%

**Table 4:** Adverse events of ADA treatment.

Trail	Experimental		Control	
	Event	Total	Event	Total
Colombel et al.	59	70	38	47
Dewint et al.	34	39	31	34
